# Mild Cognitive Impairments Attenuate Prefrontal Cortex Activations during Walking in Older Adults

**DOI:** 10.3390/brainsci10070415

**Published:** 2020-07-01

**Authors:** Roee Holtzer, Meltem Izzetoglu

**Affiliations:** 1 Ferkauf Graduate School of Psychology, Yeshiva University Bronx, New York, NY 10461, USA; 2 Department of Neurology, Albert Einstein College of Medicine Bronx, Roee Holtzer, 1225 Morris Park Avenue, Van Etten Building, New York, NY 10461, USA; 3 Department of Electrical and Computer Engineering, Villanova University, Villanova, PA 19085, USA; meltem.izzetoglu@villanova.edu

**Keywords:** MCI, fNIRS, walking, prefrontal cortex, neural efficiency

## Abstract

The presence of Mild Cognitive Impairments (MCI) is associated with worse gait performance. However, the effect of MCI on cortical control of gait, as assessed during active walking, is unknown. We hypothesized that MCI would be associated with attenuated activations and limited improvement in efficiency in the Prefrontal cortex (PFC) under cognitively-demanding walking conditions. Functional Near-Infrared Spectroscopy (fNIRS) was used to assess Oxygenated Hemoglobin (HbO_2_) in the PFC during Single-Task-Walk (STW), cognitive interference (Alpha) and Dual-Task-Walk (DTW) conditions. Three repeated trials in each experimental condition were administered. Healthy control (*n* = 71; mean age = 76.82 ± 6.21 years; %female = 50.7) and MCI (*n* = 11; mean age = 78.27 ± 4.31 years; %female = 45.5) participants were included. The increase in HbO_2_ from STW to DTW was attenuated among MCI participants compared to controls (estimate = 0.505; *p* = 0.001). Whereas, among controls, HbO_2_ increased from Alpha to DTW, the opposite was observed among MCI participants (estimate = 0.903; *p* < 0.001). In DTW, the decline in HbO_2_ from trial 1 to 2 was attenuated in MCI participants compared to controls (estimate = 0.397; *p* = 0.008). Moreover, whereas HbO_2_ declined from trial 1 to 3 among controls, MCI participants showed the opposite trend (estimate = 0.946; *p* < 0.001). MCI was associated with attenuated brain activation patterns and compromised ability to improve PFC efficiency during dual-task walking.

## 1. Introduction

Mild Cognitive Impairments (MCI) is an established risk factor for Alzheimer’s dementia [1]. The presence of MCI is determined via established diagnostic procedures requiring evidence for objective cognitive impairment [2] based on performance cutoff scores on a neuropsychological tests [3]. The development of relatively minor declines in instrumental activities of daily living due to cognitive impairments is also used to support the diagnosis of MCI [2,4]. MCI is associated with reduced grey matter volume in the whole brain, entorhinal cortex, and hippocampus (for review and meta-analysis see [5]). Lower white matter integrity, evaluated using diffusion tensor imaging (DTI), has also been documented in patients with MCI [6]. Furthermore, structural and functional brain networks in MCI are both disrupted and associated with compromised cognitive performance [7]. A recent review using functional-Near-Infrared Spectroscopy (fNIRS) revealed reduced task-related oxygenation in the frontal cortex in patients with MCI, which was further reduced in those diagnosed with dementia [8].

MCI is associated with worse gait performance [9], notably under cognitively demanding conditions such as dual-task walking (for review and meta-analysis, see [10]). Structural neuroimaging studies revealed that grey matter atrophy [11,12,13,14] and poor white matter integrity [15] were related to worse gait performance in patients with MCI. To our knowledge, however, no studies to date have examined whether MCI moderate changes in brain function during active walking. The paucity of research is, in part, attributed to the limitations of traditional neuroimaging methods requiring participants to be in a horizontal position and immobile during scanning procedures [16]. In recent years, functional Near-Infrared Spectroscopy (fNIRS) has been used to investigate changes in the hemodynamic response during active walking in humans (for reviews, see [17,18]). Specifically, studies revealed that, in older adults, fNIRS-derived Oxygenated Hemoglobin (HbO_2_) in the Prefrontal Cortex (PFC) was increased in Dual-Task-Walk (DTW) compared to Single-Task-Walk (STW) conditions due to the greater attention and executive demands imposed by DTW [19,20,21,22,23]. Moreover, repeated training trials during one experimental session resulted in improved behavioral performance and brain efficiency, the latter operationalized using fNIRS-derived HbO_2_, in DTW but not STW conditions [24].

The current study addressed an important gap in the literature. Specifically, utilizing Capacity Limitations [25,26] as a conceptual framework, we evaluated the effect of MCI on fNIRS-derived HbO_2_ in the PFC during and over repeated trials of active walking under STW and DTW conditions. Taking into account behavioral performance, we hypothesized that patients with MCI would demonstrate a failure to ramp up fNIRS-derived HbO_2_ in response to increased cognitive demands in DTW as compared to the single task conditions. We further hypothesized that, compared to healthy controls, participants with MCI would demonstrate reduced ability to improve the efficiency of their brain response (i.e., reduced fNIRS-derived HbO_2_ over repeated trials coupled with improved behavioral performance) under DTW after within session practice.

## 2. Materials and Methods

### 2.1. Participants

Participants were a subsample enrolled in “Central Control of Mobility in Aging” (CCMA) who, in addition to receiving the regular study testing procedures, had also completed a protocol that involved repeated walking trials under single and dual-task conditions while being monitored with fNIRS to assess task-related changes in the hemodynamic signal in the PFC [24,27]. Potential CCMA participants were identified from publicly available population lists that were stratified based on residential zip code and age. Trained research assistants contacted potential participants and first obtained their verbal assent to participate in a structured telephone interview to determine whether they met the initial study eligibility criteria. Once assent was obtained, initial eligibility was determined using cognitive screens and self-report measures assessing mobility function as well as psychological and medical history. Participants who provided verbal assent and passed the telephone interview were invited to two in-person annual study visits that were scheduled approximately two weeks apart. Study procedures included neuropsychological tests, objective mobility assessments, structured interviews assessing medical and psychological history as well as a range of questionnaires designed to evaluate multiple domains of function [28,29]. The training protocol involving repeated walking trials under single and dual-task condition, while being imaged with fNIRS, was completed in one session. Exclusion criteria included the following: inability to ambulate independently, dementia, current or history of severe neurological or psychiatric disorders, significant impairments in vision and/or hearing, and recent or anticipated surgeries that could affect walking. The study was in compliance with the Code of Ethics of the World Medical Association (Declaration of Helsinki). Written informed consent was obtained from each participant in the first study visit. The Institutional Review Board of Albert Einstein College of Medicine approved this study (IRB protocol # 2010-224).

### 2.2. Mild Cognitive Impairments (MCI) Status

An interdisciplinary team that included a licensed neuropsychologist, physician, and doctoral psychology trainees determined cognitive status (Normal, MCI, dementia) using established consensus diagnostic case conference procedures [30]. As previously described [31,32], a positive MCI status was determined using published criteria [3,4], which included the following: (a) The presence of objective cognitive impairment that was determined by comparing performance on neuropsychological tests to published norms; (b) mild declines on instrumental activities of daily living; (c) absence of dementia. Questionnaires with established psychometric properties were used to evaluate the presence of cognitive complaints [33,34]. The neuropsychological testing battery assessed the following cognitive domains: premorbid function, attention, visual spatial, memory (verbal and visual), language, and executive functions. 

### 2.3. Dual-Task Walking Protocol

There were two single task conditions. Under the STW condition, participants were instructed to walk around the electronic walkway at their “normal pace” for three consecutive loops. Under the Alpha (cognitive interference task) condition, participants were instructed to recite alternate letters of the alphabet (A, C, E…) for 30 seconds out loud while standing. Under the DTW condition, participants were instructed to walk around the walkway at their “normal pace” while reciting alternate letters of the alphabet for three consecutive loops. In order to reduce task prioritization effects, participants were specifically told to pay equal attention to both tasks [35,36]. To minimize task order effects, the three experimental conditions were presented in a counterbalanced order using a Latin-square design. Three repeated trials with each trial consisting of the three task conditions were administered. Participants were given five minutes to rest between trials. [24,27]. The dual-task walking protocol utilized in the current investigation has excellent reliability and validity [28,37].

### 2.4. Quantitative Gait Assessment

Quantitative measures of gait performance under the STW and DTW conditions were assessed using a 4 × 20 foot Zeno electronic walkway (Zenometrics, LLC; Peekskill, NY, USA). Entry and exit points for both task conditions were determined algorithmically using ProtoKinetics Movement Analysis Software technology (PKMAS, 5.07) [37]. Internal consistency of quantitative gait measures, assessed under both STW and DTW conditions, was excellent, as determined by >0.95 split-half intra-class correlations [19].

### 2.5. fNIRS System

Validation of the device used in the current investigation (fNIRS Imager 1100; fNIR Devices, LLC, Potomac, MD, USA) has been established in prior research [38,39]. Data collection is at a sampling rate of 2 Hz. There were four LED light sources and 10 photodetectors in the fNIRS sensor, which was placed on the forehead using a standard sensor placement procedure [40]. The fNIRS sensor included 16 voxels, with a source-detector separation of 2.5 cm. The sensor’s light sources (Epitex Inc., Kyoto, Japan; type L4X730/4X805/4X850-40Q96-I) contained three built-in LEDs (peak wavelengths at 730, 805, and 850 nm with an overall outer diameter of 9.2 ± 0.2 mm. The photodetectors (Bur Brown, Tucson, AZ, USA; type OPT101) are monolithic photodiodes with a single supply transimpedance amplifier. 

### 2.6. Preprocessing and Hemodynamic Signal Extraction

First, individual data from all optodes were visually inspected to identify and eliminate saturation, dark current conditions, or extreme noise. Wavelet denoising with Daubechies 5 (db5) wavelet was then applied to the raw intensity measurements at 730 and 850 nm wavelengths for spiky noise suppression [41]. The modified Beer-Lambert law (MBLL) was used to calculate changes in oxygenated hemoglobin (HbO_2_) and deoxygenated hemoglobin (Hb) from the artifact-removed raw intensity measurements [42]. In MBLL, we used the previously published wavelength and chromophore dependent molar extinction coefficients (ε) by Prahl, and age and wavelength adjusted differential pathlength factor (DPF) [42,43,44]. To remove possible baseline shifts and suppress physiological artifacts such as respiration and Mayer waves, we first applied Spline filtering [45] followed by a finite impulse response low-pass filter with cut-off frequency at 0.08 Hz [43]. We used HbO_2_ and not Hb as a surrogate for neural activity in the PFC because the former is more reliable and sensitive to gait-related changes in brain activation patterns [46,47]. We administered proximal 10-s baselines immediately before each experimental condition to quantify relative changes in HbO_2_ for each task [19,20,22,23,48]. A central “hub” computer with E-Prime 2.0 software synchronized the acquisition of Gait and fNIRS events [19,20,21,22,23,24]. The internal consistency of HbO_2_ measurements for each experimental conditions was excellent as determined by split-half intra-class correlations that exceeded 0.80 [19].

### 2.7. Covariates

Age, gender, Global Health Status (GHS), and depressive symptoms were used as covariates. The GHS summary score (range 0–10) included the following conditions: diabetes, chronic heart failure, arthritis, hypertension, depression, stroke, Parkinson’ s disease, chronic obstructive lung disease, angina, and myocardial infarction [30]. Depressive symptoms were assessed using the geriatric depression scale (GDS) [49]. The Repeatable Battery for the Assessment of Neuropsychological Status (RBANS) [50] was utilized to characterize cognitive function but was not included in statistical models due to overlap with the diagnosis of MCI. 

### 2.8. Statistical Analysis

The distributions of all study measures were carefully inspected. Descriptive statistics for the study outcomes and covariates were tabulated for the entire sample and stratified by MCI status. Separate linear mixed effects models (LME) were used to examine the effect of task on study outcomes. The first model evaluated the effect of task as a three-level repeated measures variable (STW, Alpha, DTW) on the change in fNIRS-derived HbO_2_ across task conditions. Separate LMEs examined the effects of task on gait velocity (two-level repeated measures; STW vs. DTW) and the rate of error in letter generation (two-level repeated measures; Alpha vs. DTW). The rate, instead of the total number of errors, was used because while the time was fixed at 30-sec for Alpha, it varied in DTW depending on the participants’ gait speed. Separate LMEs were used to determine the effects of repeated trials, stratified by task conditions, on study outcomes (HbO_2_, gait velocity, rate of error in letter generation). The moderating effects of MCI on the change in study outcomes across repeated trials were examined via two-way interactions of group status (MCI vs. Control) x trials. SPSS statistical software package, (version 25; SPSS, Inc., Chicago, IL, USA) was used for statistical analysis and *p*-values were considered significant at *p* < 0.05.

## 3. Results

### 3.1. Sample Characteristics

A cohort of 82 older adults (mean age = 77.01 ± 5.99 years; mean education = 14.84 ± 2.94 years; %female = 50) participated in the current study. The sample was relatively healthy (GHS mean score = 0.83 ± 1.06) and in the average range of overall cognitive function (RBANS mean Index score = 94.10 ± 11.45). There were 71 cognitively normal participants and 11 diagnosed with MCI. Demographic, behavioral, and fNIRS data were summarized for the study cohort and stratified by cognitive status (MCI vs. Control) in Table 1. 

### 3.2. MCI Effects: fNIRS-Derived HbO_2_ across Task Conditions

**Figure 1:**
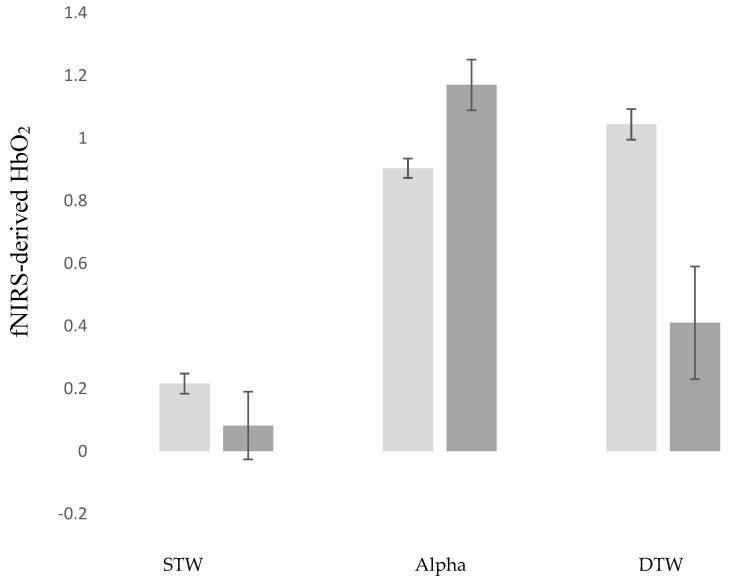
fNIRS-Derived HbO_2_ changes across task conditions in MCI and control participants. Light Grey: Control Participants; Dark Grey: MCI Participants. STW: Single-Task-Walk; Alpha: Cognitive Interference Task; DTW: Dual-Task-Walk; MCI: Mild Cognitive Impairments.

The first LME revealed that fNIRS-derived HbO_2_ was greater in DTW compared to both STW (estimate = −0.829; *p* < 0.001; 95%CI = −0.927 to −0.731) and Alpha (estimate = −0.139; *p* = 0.005; 95%CI = −0.237 to −0.041). Positive MCI status was associated with lower HbO_2_ in DTW (estimate = −0.557; *p* = 0.026; 95%CI = −1.046 to −0.067). MCI moderated the effect of task on HbO_2_. Specifically, the increase in HbO_2_ in from STW to DTW was attenuated among participants with MCI compared to controls (estimate = 0.505; *p* = 0.001; 95%CI = 0.203 to 0.807). Moreover, whereas, among controls, HbO_2_ increased from Alpha to DTW, the opposite trend was observed among MCI participants (estimate = 0.903; *p* < 0.001; 95%CI = 0.601 to 1.205). These findings were visually depicted in Figure 1. 

### 3.3. MCI Effects: fNIRS-Derived HbO_2_ across Repeated Trials 

In STW, the decline in HbO_2_ trial 1 to 3 was greater among participants with MCI compared to controls (estimate = −0.541; *p* < 0.001). All other effects were not significant (see model 1 in Table 2 for details regarding this analysis). 

In Alpha, fNIRS-derived HbO_2_ declined from trial 1 to 2 (estimate = −0.285; *p* < 0.001) and from Trial 1 to 3 (estimate = −0.201; *p* < 0.001). MCI status moderated the effect of repeated trials on HbO_2_. Specifically, whereas among controls HbO_2_ declined from trial 1 to 2, participants with MCI showed the opposite trend (estimate = 0.320; *p* = 0.002). Details of this analysis are summarized in model 2 in Table 2. 

**Figure 2:**
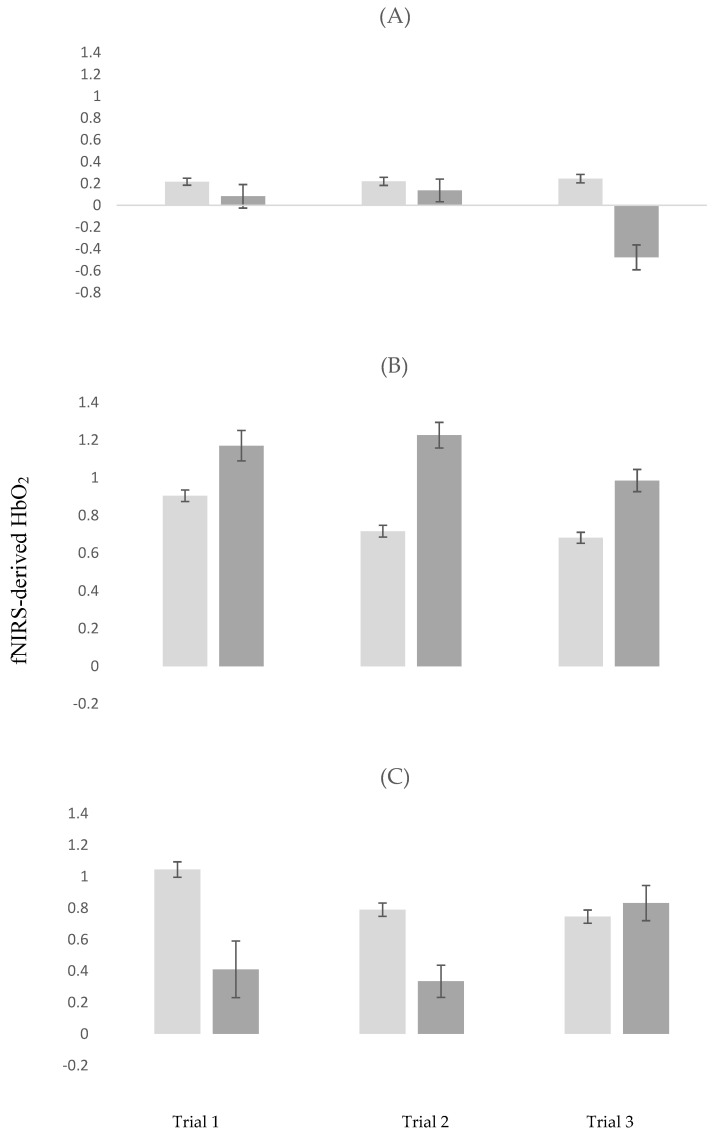
Changes in fNIRS-derived HbO_2_ across repeated trials stratified by task conditions in MCI and control participants. Light Grey: Control Participants; Dark Grey: MCI Participants; (**A**) repeated trials in STW (Single-Task-Walk); (**B**) repeated trials in Alpha (Cognitive Interference Task); (**C**) repeated trials in DTW (Dual-Task-Walk); MCI: Mild Cognitive Impairments.

In DTW, fNIRS-derived HbO_2_ declined from trial 1 to 2 (estimate = −0.312; *p* < 0.001) and from Trial 1 to 3 (estimate = −0.345; *p* < 0.001). MCI status moderated the effect of repeated trials on HbO_2_. Specifically, the decline in HbO_2_ from trial 1 to 2 was attenuated in participants with MCI compared to controls (estimate = 0.397; *p* = 0.008). Moreover, whereas HbO_2_ declined from trial 1 to 3 among controls, participants with MCI showed the opposite trend (estimate = 0.946; *p* < 0.001). Details of this analysis are summarized in model 3 in Table 2. The moderating effects of MCI on HbO_2_ across repeated trials, stratified by task condition, are visually depicted in Figure 2A–C.

### 3.4. MCI Effects: Behavioral Outcomes across Task Conditions

#### 3.4.1. Gait Velocity

LME revealed that, as expected, gait velocity declined from STW to DTW (estimate = −11.154; *p* < 0.001; 95%CI = −14.348 to −7.960). Gait velocity was slower among participants with MCI though this effect was not significant (estimate = −11.087; *p* = 0.197; 95%CI = −28.059 to 5.884). The interaction of task x group status was not significant (estimate = 0.879; *p* = 0.850; 95%CI = −8.341 to 10.100). 

#### 3.4.2. Rate of Errors in Letter Generation

LME revealed that, as expected, the rate of errors in letter generation increased from Alpha to DTW (estimate = −11.154; *p* < 0.001; 95%CI = −14.348 to −7.960). The rate of errors in letter generation was greater among participants with MCI compared to controls (estimate = 0.474; *p* = 0.010; 95%CI = 0.115 to 0.833). The interaction of task x group status was not significant (estimate = −0.219; *p* = 0.530; 95%CI = −0.914 to 0.474). 

### 3.5. MCI Effects: Behavioral Outcomes across Repeated Trials

#### 3.5.1. Gait Velocity

In STW, gait velocity marginally increased from trial 1 to 2 (estimate = 1.023; *p* = 0.049). Compared to controls, participants with MCI had slower gait velocity in trial 3 (estimate = −11.425; *p* = 0.029). All other effects were not significant (see model 1 in Table 3 for details regarding this analysis). 

**Figure 3:**
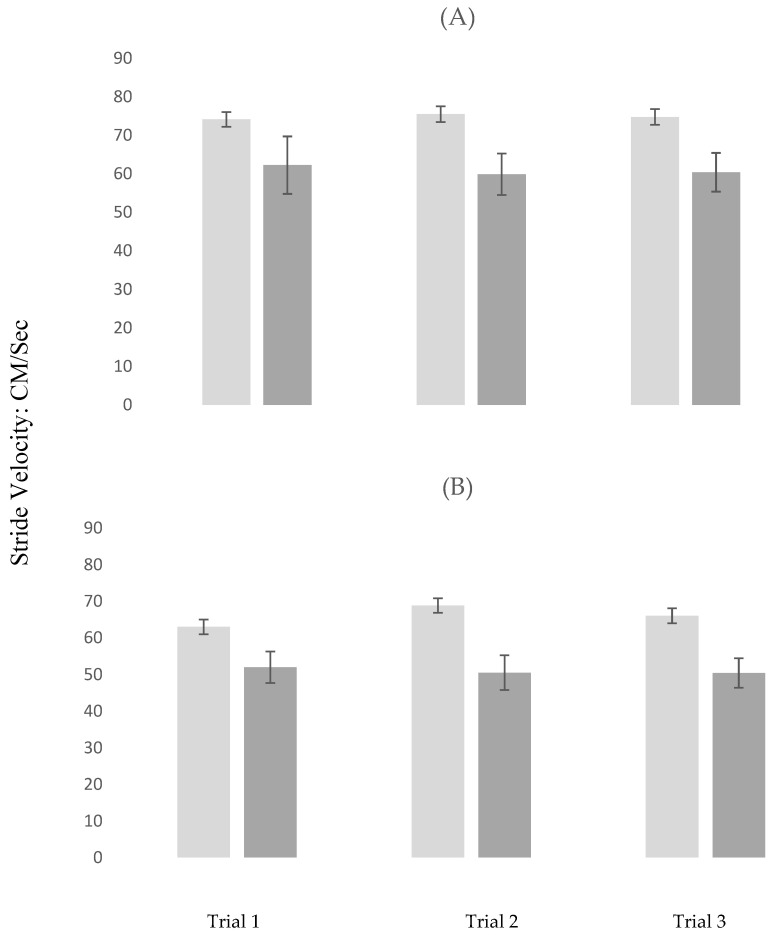
Changes in gait velocity across repeated trials stratified by task conditions in MCI and control participants. Light Grey: Control Participants; Dark Grey: MCI Participants; (**A**) repeated Trials in STW (Single-Task-Walk); (**B**) repeated Trials in DTW (Dual-Task-Walk); MCI = Mild Cognitive Impairments.

In DTW, gait velocity increased from trial 1 to 2 (estimate = 2.154; *p* < 0.001) and from Trial 1 to 3 (estimate = 2.664; *p* < 0.001). Compared to controls, participants with MCI had slower gait velocity in trial 3 (estimate = −10.671; *p* = 0.040). MCI status moderated the effect of repeated trials on gait velocity. Specifically, the increase in gait velocity from trial 1 to 2 was attenuated in participants with MCI compared to controls (estimate = −3.545; *p* = 0.038) and from trial 1 to 3 (estimate = −4.141; *p* < 0.001). Details of this analysis are summarized in model 2 in Table 3. The moderating effects of MCI on gait velocity across repeated trials are visually depicted, stratified by task, in Figure 3A,B.

#### 3.5.2. Rate of Errors in Letter Generation

In Alpha, the rate of errors in letter generation declined from trial 1 to 2 (estimate = −0.121; *p* < 0.026). The presence of MCI was associated higher rate of errors in letter generation in trial 1 (estimate = 0.391; *p* = 0.029). MCI status moderated the effect of repeated trials on the rate of errors in letter generation. Specifically, compared to controls, participants with MCI showed a greater decline from trial 1 to 3 (estimate = −0.538; *p* = 0.003). Details of this analysis are summarized in model 1 in Table 4.

**Figure 4:**
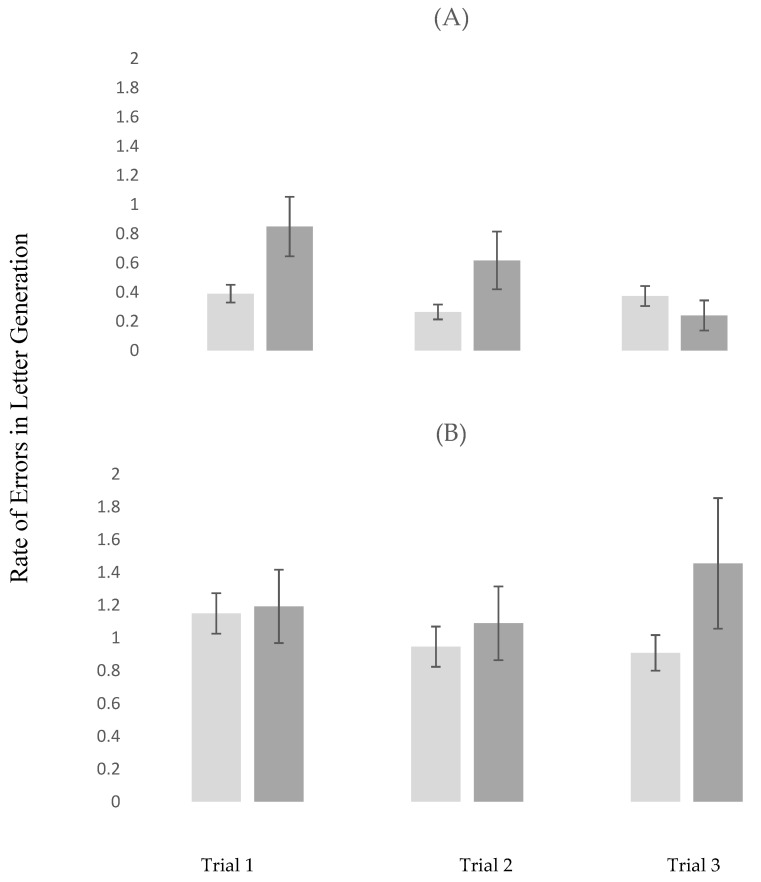
Changes in errors in letter generation across repeated trials stratified by task conditions in MCI and control participants. Light Grey: Control Participants; Dark Grey: MCI Participants; (**A**) repeated trials in Alpha (Cognitive Interference Task); (**B**) repeated trials in DTW (Dual-Task-Walk); MCI = Mild Cognitive Impairments.

In DTW, the rate of errors in letter generation increased from trial 1 to 3 (estimate = 0.261; *p* < 0.012). The remaining effects, which were not significant, were detailed in model 2 in Table 4. The moderating effects of MCI on the rate of errors in letter generation across repeated trials, stratified by task, are visually depicted in Figure 4A,B.

## 4. Discussion

The present investigation determined the moderating effect of MCI on changes in fNIRS-derived HbO_2_ in the PFC, as assessed during active walking, across conditions, and repeated trials that experimentally manipulated cognitive demands. We found that participants with MCI demonstrated attenuated increases and even a decline in the hemodynamic response in the PFC from single to dual-task conditions. Furthermore, the presence of MCI was associated with differential changes in activations patterns over repeated trials within tasks as compared to healthy controls. These findings are discussed below.

This study, to our knowledge, is the first to report on the effect of MCI on the hemodynamic response in the PFC during active walking. Confirming the first hypothesis of the study, we found that, compared to controls, the increase in fNIRS-derived HbO_2_ from STW to DTW was attenuated among participants with MCI. Moreover, fNIRS-derived HbO_2_ declined from Alpha to DTW among participants with MCI, whereas healthy older adults showed the opposite pattern with the expected increase in activation in the PFC in response to the greater cognitive demands imposed by DTW compared to the single tasks. This finding is consistent with our hypothesis and conceptual framework of capacity limitation [25,26]. Specifically, capacity limitation proposes that neural under-activations in response to cognitive challenges that increase in terms of complexity and difficulty result from injury or damage to structural and functional brain systems that sub-serve task-specific cognitive demands. The literature established strong evidence for damage to structural brain regions implicated in cognitive [7] and walking [11,12,13,14] performance in MCI. A previous fMRI study reported a task-specific under-activation patterns in patients with MCI compared to healthy controls in a network consisting of occipitotemporal regions and inferior frontal cortex. Moreover, reduced fMRI response among patients with MCI in the left and right midfusiform gyri accurately discriminated them from healthy controls [51]. A review of fNIRS studies reported that, compared to controls, patients with MCI demonstrated reduced oxygenation in both resting states and several functional tasks, notably in the frontal cortex [8]. Hence, the findings reported herein are consistent with and incremental to the current literature extending our understanding of the attenuating effect of MCI on brain function to PFC regulation of attention-demanding locomotion. 

Positive MCI status was associated with different patterns of change in fNIRS-derived HbO_2_ across repeated trials of different task conditions. The findings confirm the second study hypothesis, indicating that participants with MCI would demonstrate compromised ability to improve the efficiency of their brain response and behavioral performance after within session practice. Neural inefficiency is defined in the context of behavioral or cognitive outcomes and is operationalized as higher neural activation that is coupled with similar or worse performance, suggesting the brain has to expend more energy to support function [52]. Here, healthy older adults demonstrated improved performance that was coupled with declines in fNIRS-derived HbO_2_ over repeated DTW trials. This pattern of results suggests that task-related PFC efficiency was improved due to practice. These findings are consistent with our previous work [24,27] and a metanalytic fMRI review study [53] suggesting reduced activation due to practice on cognitively demanding tasks, notably in the PFC, a brain region involved in both novelty and learning [54]. The participants with MCI, however, demonstrated a differential pattern of activation across repeated DTW trials, as evidenced by the significant trial by group status (MCI vs. controls) interactions (see model 3 in Table 2 and Figure 2C for visual depiction). Specifically, among participants with MCI, fNIRS-derived HbO_2_ was low in trials 1 and 2 but then increased in trial 3 to reach levels similar to those exhibited by the healthy controls. fNIRS-derived HbO_2_ declined over repeated Alpha trials, but MCI and control participants showed a differential pattern of change as evidenced by the two-way interaction of group status (MCI vs. controls) by trial (see model 2 in Table 2 and Figure 2B). Specifically, whereas among healthy controls fNIRS-derived HbO_2_ declined from trials 1 to 2, the opposite trend was manifested by the MCI participants. Both groups demonstrated decline in activation in trial 3 compared to trial 1 suggesting that improved brain efficiency on this task was delayed in those diagnosed with MCI. Trial effects were not significant in STW but there was a trial by group interaction where participants with MCI showed an unexpected decline in fNIRS-derived HbO_2_ in trial 3. Careful inspection of the data did not reveal any evidence for extreme values or skewness that may have influenced this finding. Because there is no clear theoretical or empirical explanation for this finding, it might be viewed as a spurious result that, at least in part, could be attributed to the small number of MCI participants.

The above findings should be interpreted in the context of the behavioral results. Participants with MCI had slower gait velocity in both STW and DTW as compared to the healthy controls. Moreover, the group by trial interactions in DTW were significant, indicating that improvement over repeated trials in gait performance was evident in the control but not MCI participants (see model 2 in Table 3 and Figure 3B for visual depiction). In Alpha, participants with MCI demonstrated higher error rate in letter generation compared to the controls in trial 1. There was an overall significant improvement in performance from trial 1 to 2 as well as a significant interaction whereby participants with MCI showed a greater improvement in the error rate of letter generation in trial 3 compared to trial 1; this improvement brought their performance level in the third trial closer to that of their healthy counterparts. In DTW, there was a significant increase in the error rate of letter generation in the third compared to the first trial, which despite the insignificant group by trial interaction, was likely attributed to participants in the MCI group (see model 2 in Table 4 and Figure 4B for visual depiction). Integrating the neuroimaging and behavioral findings provides a few key observations that have important clinical implications. First, the effect of MCI on study outcomes was much stronger using the fNIRS compared to the behavioral data. Specifically, the group by task interactions were not significant using gait velocity or the error rate in letter generation as the outcomes, the effect sizes for these non-significant interactions (Cohen d deduced from the LMEs) were 0.020 and 0.069, respectively. In contrast, effect sizes for the moderation effects of MCI (i.e., significant group by task interactions) on the change in fNIRS-derived HbO_2_ across task conditions were significant for both STW vs. DTW (d = 0.36) and Alpha vs. DTW (0.64). Moreover, while both significant, effect sizes for the moderation effects of MCI on the change across DTW trials were larger for fNIRS-derived HbO_2_ (Cohen d = 0.29 for trial 1 vs. trial 2 and Cohen d = 0.69 for trial 1 vs. trial 3) than gait velocity (Cohen d = 0.23 for trial 1 vs. trials 2 and 3). Hence, it appears that, compared to the behavioral data, fNIRS-derived HbO_2_ was more sensitive to MCI status. Second, the heathy control but not MCI participants showed improved performance and PFC efficiency in DTW, which is cognitively more demanding as compared to the single tasks. Previous research revealed that poor DTW performance predicted incident frailty, disability, and mortality [55] and that inefficient PFC activation during DTW was associated with increased risk of incident falls among relatively healthy older individuals who resided in the community [56]. Hence, while DTW may be an attractive target for training, it appears that cognitive status is important to consider when designing interventions to improve mobility function. While training in dual-task walking may be beneficial for healthy older adults, it seems that those with MCI might benefit from training on the single tasks first. It is noteworthy that Alpha performance was improved by the third trial in participants with MCI. 

The number of MCI participants in the current study was small. Hence, the reliability and generalizability of these findings should be examined in future studies with larger MCI cohorts that will also afford stratification by MCI subtypes. Traditional neuroimaging (e.g., MRI) was not included in the current study, limiting further insights into potential mechanisms that may explain the under activation observed in MCI participants. A recent review emphasized the importance of examining brain structure and function relations in aging and MCI using MRI methods [57]. Previous work demonstrated that white matter integrity [58] and grey matter volume [59] moderated fNIRS-derived HbO_2_ levels assessed during active walking in participants aged 65 or older. It would, therefore, be of importance to examine whether the structural integrity of the brain also influences the changes in fNIRS-derived HbO_2_ due to walking conditions and training in participants with MCI. A recent study provided evidence that using multiple filters as well as age and wavelength adjusted differential pathlength factor (DPF) to process fNIRS data of locomotion yielded similar outcomes in terms of the effect of task on both HbO_2_ and Hb [42]. The current study utilized a stringent approach to data processing by applying the combined filters and DPFs described by Izzetoglu and Holtzer [42] as well as filters specifically designed to remove motion artifacts to protect the validity of the study outcomes. Given that task effects on both HbO_2_ and Hb have been reported in multiple previous publications [42] and in order to reduce the possibility of false discovery rate, we elected not to include Hb as an additional outcome measure in the current investigation. 

## 5. Conclusions

We provided preliminary evidence that brain activation in the PFC was attenuated under attention-demanding walking conditions among older persons with MCI. Moreover, individuals with MCI demonstrated compromised the ability to improve performance and PFC efficiency after within session practice of dual-task walking. 

## Figures and Tables

**Table 1:** Descriptive Statistics of the Study Sample.

Number (%) Female	Total Sample (*n* = 82) 41 (50)	Control (*n* = 71) 36 (50.7)	MCI (*n* = 11) 5 (45.5)
Variable	*M* (*SD*)	*M* (*SD*)	*M* (*SD*)
Age	77.01 (5.99)	76.82 (6.21)	78.27 (4.31)
GHS	0.83 (1.06)	0.83 (1.08)	0.82 (0.98)
Education	14.84 (2.94)	15.00 (2.86)	13.82 (3.40)
RBANS Total:	94.10 (11.45)	95.58 (11.00)	84.55 (9.96)
RBANS Index Scores			
Immediate Memory	102.48 (12.69)	103.65 (11.70)	94.91 (16.54)
Visual/Spatial/Construction	90.68 (11.74)	91.32 (11.62)	86.55 (12.29)
Attention	101.53 (14.13)	102.85 (13.84)	91.11 (12.56)
Language	94.58 (9.53)	95.41 (9.61)	89.27 (7.33)
Delayed Memory	96.48 (11.21)	97.28 (11.28)	91.27 (9.62)
GDS	4.40 (3.35)	4.32 (3.30)	4.91 (3.78)
Stride Velocity STW	72.71 (16.79)	74.14 (15.55)	62.24 (22.39)
Stride Velocity DTW	61.66 (16.31)	62.99 (16.36)	51.96 (12.83)
Error Letter Generation: Alpha	0.44 (0.54)	0.38 (0.51)	0.84 (0.61)
Error Letter Generation: DTW	1.15 (0.97)	1.14 (1.01)	1.19 (0.59)
HbO_2_ STW	0.20 (1.00)	0.21 (0.98)	0.08 (1.12)
HbO_2_ Alpha	0.93 (0.93)	0.90 (0.94)	1.17 (0.83)
HbO_2_ DTW	0.97 (1.54)	1.04 (1.49)	0.41 (1.84)

GHS: global health score; RBANS: repeatable battery for the assessment of neuropsychological status; GDS: Geriatric Depression Scale; Error Letter Generation: rate of errors per task completion time; STW: single-task walk; DTW: dual-task-walk; HbO_2_: Oxygenated Hemoglobin.

**Table 2:** Linear-Mixed-Model Estimates of Trial Effects on fNIRS-Derived HbO_2_ in STW, Alpha and DTW.

	Model 1: Trial Effects, STW			Model 2: Trial Effects, Alpha			Model 3: Trial Effects, DTW		
Variable	Estimate	95% CI	*p*	Estimate	95% CI	*p*	Estimate	95% CI	*p*
Trial 1 vs. Trial 2	−0.001	(−0.057, 0.110)	0.535	−0.218	(−0.285, −0.151)	<0.001	−0.312	(−0.409, −0.215)	<0.001
Trial 1 vs. Trial 3	−0.001	(−0.084, 0.082)	0.998	−0.201	(−0.269, −0.134)	<0.001	−0.345	(−0.452, −0.256)	<0.001
MCI	0.014	(−0.497, 0.525)	0.956	0.085	(−0.368, 0.538)	0.709	−0.654	(−1.319, 0.009)	0.053
MCI x Trial 1 vs. Trial 2	0.101	(−0.149, 0.352)	0.429	0.320	(0.117, 0.522)	0.002	0.397	(0.104, 0.690)	0.008
MCI x Trial 1 vs. Trial 3	−0.541	(−0.795, −0.286)	<0.001	0.056	(−0.148, 0.261)	0.589	0.946	(0.650, 1.243)	<0.001
Age	−0.022	(−0.049, 0.004)	0.102	0.003	(−0.020, 0.227)	0.758	−0.015	(−0.050, 0.020)	0.399
GHS	0.011	(−0.150, 0173)	0.887	0.077	(−0.066, 0.222)	0.287	0.066	(−0.146, 0.278)	0.536
Gender	0.057	(−0.282, 0.397)	0.736	−0.302	(−0.606, 0.002)	0.052	−0.157	(−0.603, 0.289)	0.486
GDS	<0.001	(−0.054, 0.054)	0.998	<0.001	(−0.049, 0.048)	0.993	−0.041	(−0.1139, 0.030)	0.252

STW: Single-Task-Walk; Alpha: Cognitive Interference Task; DTW: Dual-Task-Walk; HbO_2_: Oxygenated Hemoglobin; GHS: Global Health Score; GDS: Geriatric Depression Scale; MCI: Mild Cognitive Impairments.

**Table 3:** Linear-Mixed-Model Estimates of Trial Effects on Gait Velocity in STW and DTW.

	Model 1: Trial Effects, STW			Model 2: Trial Effects, DTW		
Variable	Estimate	95% CI	*p*	Estimate	95% CI	*p*
Trial 1 vs. Trial 2	1.023	(0.006, 2.040)	0.049	2.154	(0.980, 3.328)	<0.001
Trial 1 vs. Trial 3	0.570	(−0.730, 1.871)	0.385	2.664	(1268, 4.060)	<0.001
MCI	−11.425	(−21.675, −1.74)	0.029	−10.671	(−20.823, −0.51)	0.040
MCI x Trial 1 vs. Trial 2	−2.045	(−4.945, 0.854)	0.164	−3.545	(−6.883, −0.206)	0.038
MCI x Trial 1 vs. Trial 3	−1.067	(−4.702, 2.566)	0.560	−4.141	(−8.055, −0.227)	0.038
Age	−0.932	(−1.509, −0.354)	0.002	−0.489	(−1.076, 0097)	0.101
GHS	−4.082	(−7.518, −0.646)	0.021	−2.982	(−6.466, 0.501)	0.092
Gender	5.528	(−1.594, 12.652)	0.126	−1.254	(−8.495, −5.598)	0.731
GDS	−0.359	(−1.438, 0.718)	0.509	−0.153	(−1.2367, 0.930)	0.779

STW: Single-Task-Walk; Alpha: Cognitive Interference Task; DTW: Dual-Task-Walk; GHS: Global Health Score; GDS: Geriatric Depression Scale; MCI = Mild Cognitive Impairments.

**Table 4:** Linear-Mixed-Model Estimates of Trial Effects on Rate of Error Letter Generation in Alpha and DTW.

	Model 1: Trial Effects, Alpha			Model 2: Trial Effects, DTW		
	Estimate	95% CI	*p*	Estimate	95% CI	*p*
Trial 1 vs. Trial 2	−0.121	(−0.227, −0.015)	0.026	−0202	(−0.415, 0.010)	0.062
Trial 1 vs. Trial 3	−0.030	(−0.151, 0.090)	0.619	0.261	(−0.463, −0.058)	0.012
MCI	0.391	(0.040, 0.743)	0.029	0.320	(−0.434, 1.0743)	0.400
MCI x Trial 1 vs. Trial 2	−0.081	(−0.393, 0.230)	0.604	−0.158	(−0.823, 0.507)	0.637
MCI x Trial 1 vs. Trial 3	−0.538	(−0.883, −0.192)	0.003	0.266	(−0.365, 0.899)	0.403
Age	0.014	(−0.001, 0.030)	0.077	0.015	(−0.017, 0.048)	0.341
GHS	0.008	(−0.087, 0.104)	0.866	0.055	(−0.137, 0.247)	0.570
Gender	−0.130	(−0.331, 0.069)	0.197	−0.394	(−0.802, 0.013)	0.058
GDS	−0.001	(−0.030, 0.029)	0.988	−0.019	(−0.080, 0.0040)	0.513

Alpha: Cognitive Interference Task; DTW: Dual-Task-Walk; GHS: Global Health Score; GDS: Geriatric Depression Scale; MCI: Mild Cognitive Impairments.
